# Porvoo sarcopenia and nutrition trial: effects of protein supplementation on functional performance in home-dwelling sarcopenic older people - study protocol for a randomized controlled trial

**DOI:** 10.1186/1745-6215-14-387

**Published:** 2013-11-14

**Authors:** Mikko P Bjorkman, Merja H Suominen, Kaisu H Pitkälä, Harriet U Finne-Soveri, Reijo S Tilvis

**Affiliations:** 1 Geriatric Unit, Department of Internal Medicine, University of Helsinki, PO Box 340, 00029 HUS Helsinki, Finland; 2 Society for Memory Disorders Expertise in Finland, Fredriksberginkatu 2, 00240 Helsinki, Finland; 3 Unit of General Practice, Helsinki University Central Hospital, Department of General Practice, University of Helsinki, PO Box 20, 00014 Helsinki, Finland; 4 National Institute for Health and Welfare, P.O. Box 30, FI-00271 Helsinki, Finland

**Keywords:** Sarcopenia, Frailty, Cachexia, Older people, Community-dwelling, Randomized controlled trial

## Abstract

**Background:**

Age-related muscle loss (that is, sarcopenia) is a common health problem among older people. Physical exercise and dietary protein have been emphasized in prevention and treatment of sarcopenia. Rigorous trials investigating the effects of protein supplementation on physical performance in sarcopenic populations are still scarce. The aim of this study is to investigate the effects of protein supplementation along with simple home-based exercises on physical performance among home-dwelling sarcopenic older people.

**Methods/Design:**

During 2012 the entire 75 and older population (N = 3,275) living in Porvoo, Finland was contacted via a postal questionnaire. Persons at risk of sarcopenia are screened with hand grip strength and gait speed. Poorly performing persons are further examined by segmental bioimpendance spectroscopy to determine their skeletal muscle index. Sarcopenic patients (target N = 250) will be enrolled in a 12-month randomized controlled trial with three arms: 1) no supplementation, 2) protein supplementation (20 grams twice a day), and 3) isocaloric placebo. All the participants will receive instructions on simple home-based exercises, dietary protein, and vitamin D supplementation (20 μg/d). The recruitment of patients will be completed during 2013. The primary endpoint of the trial is the change in short physical performance battery score and percentage of patients maintaining or improving their physical performance. Secondary endpoints will be, among other things, changes in muscle functions, nutritional status, body composition, cognition, quality of life, use of health care services, falls, and mortality. The assessment times will be 0, 6, 12 and 24 months.

**Discussion:**

To our knowledge, this is the first large scale randomized controlled trial among community dwelling older people with sarcopenia that focuses on the effects of protein supplementation on physical performance.

**Trial registration:**

ACTRN12612001253897, date of registration 28 October 2012, first patient was randomized 11 April 2012.

## Background

Age-related muscle loss (that is, sarcopenia, a syndrome characterized by progressive and generalized loss of skeletal muscle mass and strength [1,2]) is closely related to malnutrition, physical inactivity, inflammation, and cachexia (disease-related muscle loss) [3], which all are hallmarks of frailty [4]. All these conditions are associated with a risk of adverse outcomes (for example, physical disability, poor quality of life, and increased risk of morbidity and mortality [1-4]). According to the European consensus, sarcopenia is defined as low fat-free mass related to squared height in combination with low muscle strength or slow gait speed [1]. However, there has been practical difficulties in assessing muscle mass and, thus, diagnosing sarcopenia [2]. Several methods are available for the measurement of fat-free mass. These methods include magnetic resonance imaging, computer tomography, dual x-ray absorptiometry (DEXA), and bioimpedance analysis (BIA). The validity of conventional BIA measurements has been questioned, but recent evidence on direct segmental multifrequency measurements (DSM-BIA) has shown good accuracy in comparison with DEXA [5]. The fully portable BIA devices also allow the assessment of disabled older persons during home visits. Furthermore, changes in segmental intracellular resistance measured by bioimpedance spectroscopy (BIS), an extension of DSM-BIA, has been shown to be associated with changes in mobility in typical nursing home residents prone to sarcopenia, frailty, and cachexia [6].

Even though the mechanisms of sarcopenia are complex, adequate physical activity and nutrition are important for the maintenance of muscle functions in older people [1]. The broad health benefits of resistance exercise in older people are relatively well documented [7]. However, the lack of transportation services for the disabled patients to the exercise facilities and the lack of human resources often limit the large scale use of these regimens in clinical practice. Evidence from intervention studies shows that nutritional supplementation of older people at risk of malnutrition increases their body weight and seems to decrease the risk of death [8]. Furthermore, in addition to physical exercise, the role of dietary proteins and vitamin D has been emphasized in the prevention and treatment of sarcopenia and frailty [9-14]. According to a 2013 systematic review, nutritional supplementation is effective in improving muscle mass and to some extent muscle strength as well in old age [15]. However, long-term evidence on the effects on physical performance from representative sarcopenic populations of older people is scarce [13,14].

The aim of this randomized controlled trial is to investigate whether and to what extent protein supplementation and simple home-based exercises are able to maintain physical performance in community dwelling older people with sarcopenia. In addition, we will examine the effects of the intervention on different aspects of muscle health, quality of life, use of health-care services and mortality.

## Methods/Design

### General design

This study is a randomized controlled trial in which the community dwelling older people with sarcopenia living in Porvoo, Finland, are randomly allocated into three arms: 1) no supplementation, 2) protein supplementation, and 3) isocaloric placebo. In addition, all the participants will receive instructions on simple home-based exercises, importance of dietary protein, and the use of vitamin D supplementation with the dose of 20 μg/d. The intervention will last for 12 months. The study has been approved by the Ethics Committee of the Helsinki University Central Hospital. Informed consent will be obtained from each patient or if necessary from their closest proxy before any study procedures, which will be performed according to good clinical practice [16].

### Participants

A postal questionnaire was sent in 2012 to all people aged 75 years and older living in Porvoo, Finland (N = 3,275). The persons at risk of sarcopenia (limitations in activities of daily living, low physical activity, falls, exhaustion, high age, or low body mass index (BMI)) will be contacted by telephone, and those interested in further examinations will be screened by hand grip strength [17] and habitual gait speed [18]. Persons with low hand grip or slow habitual gait speed and low skeletal muscle index (SMI) measured by segmental calf BIS [6,19] will be recruited one by one, with researchers approaching them or their closest proxy.

Inclusion criteria and exclusion of participants:

1. 75 years or older, living permanently at home in Porvoo, Finland

2. able to walk indoors independently (canes and walkers allowed)

3. able to co-operate with hand grip, walking speed, and BIS measurements

4. low hand grip strength (men ≤30.0 kg, women ≤20.0 kg) or slow habitual gait speed (≤0.80 m/s)

5. low SMI (2 standard deviations below young adults) measured by segmental calf BIS

6. voluntary participation, written informed consent to participate in study by participant or her/his closest proxy.

7. plasma creatinine <150 μmol/l

8. no terminal illness (estimated prognosis >6 months)

9. no pacemaker

10. no bilateral replacement arthroplasty of the knee

11. no severe skin lesions in BIS electrode placement sites (dorsal foot, dorsal ankle, lateral knee, dorsal wrist, and dorsal palm)

Those persons fulfilling inclusion criteria are invited to participate. All participants are asked for an informed consent before the start of any trial procedures. In case of the participants’ cognitive decline (Mini Mental State Examination (MMSE) <19) [20] or poor capability of judgment, the proxy is invited to give consent in addition to receiving consent of the participant. The recruitment of patients will be completed during 2013.

### Study procedures

The first baseline study visit (day clinic or home visit) includes the measurement of hand grip strength, habitual gait speed, and BMI. Patients with low hand grip or slow gait speed will be further evaluated for their nutritional status. These patients will be also further examined during second baseline study visit (day clinic or home visit) including cognition, segmental BIS, physical performance, muscle power, and muscle endurance. In addition, the questionnaire data will be confirmed and deep frozen (−20 Celsius) blood samples collected.

**Figure 1:**
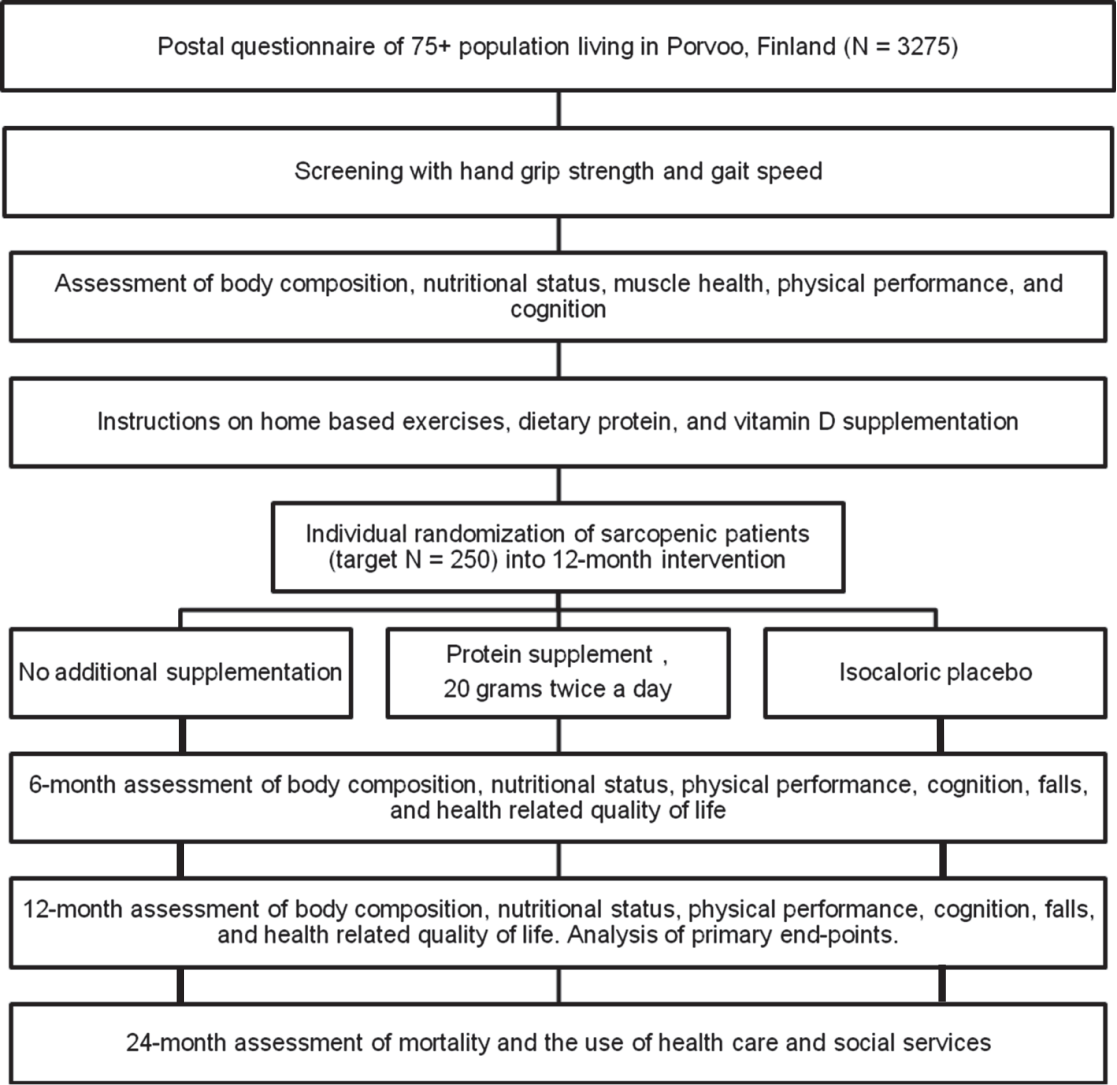
**Flow chart**[34]**.**

Two consecutive measures of handgrip strength (kg) at both hands are measured to the nearest 1.0 kg with subjects sitting in an upright position and the arm in a 90-degree angle (JAMAR dynamometer, Saehan Corp. Masan, Korea) [17]. The mean of the best result from both hands will be recorded. Habitual gait speed is measured over a 4-m course without a walking aid when possible and the best time of two attempts will be recorded to calculate the gait speed = distance (m)/time (s) [18]. SMI will be measured with a single channel, tetra polar BIS device (SFB7, ImpediMed Ltd., Eight Miles Plains, Queensland, Australia) that scans 256 frequencies between 4 kHz and 1000 kHz. Raw data are analyzed with the supporting software (version 5.3.1.1, SFB7, ImpediMed Ltd., Eight Miles Plains, Queensland, Australia) supplied by the manufacture to obtain values for the calculation of SMI (SMI = electro distance^2^/calf intracellular resistance [6,19]). In addition to the participants, SMI will be also measured in young healthy adults to provide the cut-points for low SMI values. Physical performance will be assessed by the short physical performance battery (SPPB) [21], which consists of three components: balance, gait speed, and chair rise ability. Scores of 1 to 4 are based on categories of performance in the balance tests, on the time necessary to complete the walk, and on the time needed to perform the chair-rise test. A summary performance score of 0 to 12 will be calculated by summing the scores of the tests. These three components (balance, gait speed, and chair rise) will also used to calculate continuous summary physical performance scores (CSPPS = 0 to 100) [22,23] Muscle power will be measured by the Takai *et al*. chair stand test [24] and muscle endurance by the two-minute step test [25]. Nutritional status will be assessed by the Mini Nutritional Assessment (MNA) [26], the dietary quality questionnaire [27] and the three-day dietary record [28]. Cognition will be assessed by the Clinical Dementia Rating Scale (CDR) [28], the Mini Mental State Examination (MMSE) [20], verbal fluency [29,30], and the clock drawing test [31]. Health-related quality-of-life (HrQOL) will be assessed by SF-36 [32] and 15D [33] measures. Participants will be examined by the research group three times during the year: at baseline, and at 6, and 12 months. The flow chart of the study is presented in Figure 1, and study assessment procedures are described in Table 1. Hospitalizations, use of other health and social services and death dates will be retrieved from the central registers until two years from baseline measurements.

**Table 1:** Study assessment procedures and timetable

**Assessment**	**Postal questionnaire**	**Baseline**	**6 months**	**12 months**	**24 months**
Demographics, diagnoses, drugs, supplements, exhaustion, walking aid	X				
Inclusion criteria, informed consent	X	X			
Physical activity	X	X	X	X	
15D^1^ and SF-36^2^ QOL measures	X	X	X	X	
MNA^3^, dietary quality questionnaire, dietary record		X	X	X	
BMI^4^, SMI^5^		X	X	X	
Hand grip strength (Bohannan 2008)		X	X	X	
SPPB^6^		X	X	X	
Takai chair stand test (Takai *et al*. 2009)		X	X	X	
Two-minute step test (Rikli and Jones 1999)		X	X	X	
CDR^7^		X	X	X	
MMSE^8^		X	X	X	
Verbal fluency (Morris *et al*. 1989), clock drawing test (Sunderland *et al*. 1989)		X	X	X	
Deep frozen blood samples		X	X	X	
Falls		X	X	X	
Hospitalization, use of health and social services, mortality				X	X

^1^15D = health related quality of life scale (Sintonen et al. 1990); ^2^SF-36 = health related quality of life scale (Hays *et al*. 1993); ^3^MNA = mini nutritional assessment (Guigoz *et al*. 2002); ^4^BMI = body mass index; ^5^SMI = skeletal muscle index (Björkman *et al*. 2012); ^6^SPPB = short physical performance battery (Guralnik *et al*. 1994); ^7^CDR = Clinical dementia Rating Scale (Hughes *et al*. 1982); ^8^MMSE = Minimental State Examination (Folstein *et al*. 1972). Questionnaires will be in Finnish or Swedish according to participant’s first language.

After receiving an informed consent the patients fulfilling the inclusion criteria are randomized by computer-generated random numbers (Microsoft Excel 2010, Redmond, WA, USA) into three arms: 1) no supplementation, 2) protein supplementation, and 3) isocaloric placebo. First a list including 100 sets of the numbers 1, 2, and 3 in random order is computer-generated to indicate each treatment group, which results in a list of 300 open slots with 100 slots for each treatment group. At the time of each patient recruitment a random number between 1 and 3 is computer-generated to indicate the open slot in the original list (1 = first open slot, 2 = second open slot, 3 = third open slot) to rule out the selection bias by the research group. The group treatment is randomized off-site and the content of the supplement (protein supplement versus isocaloric placebo) is kept sealed outside the research group until data collection and analysis of endpoints are complete. Furthermore, all the supplements are packed in blank 250-ml tetras and flavored with strawberry.

### Intervention

All participants receive instructions on simple home-based exercises, dietary protein and vitamin D supplementation. The home-based exercises are based on the national recommendations (http://www.voimaavanhuuteen.fi) to support walking and the role of chair, stand and step exercises are emphasized with written instructions. The participants are instructed to exercise 10 to 30 minutes twice a day. Participants are also given written information on dietary protein and nutrition in old age based on national recommendations (http://www.ravitsemuskotona.fi) as well as encouraged to use vitamin D supplementation with a dose of 20 μg/d.

The three arms of the intervention include: 1) no supplementation, 2) protein supplementation, and 3) isocaloric placebo. In groups 2 and 3 the participants are instructed to take twice daily a 250 ml beverage as a snack between meals immediately after an exercise session, whereas in group 1 the participants are instructed to take regular protein rich foods in similar fashion. In the protein supplementation group the participants will receive a maximum of 40 grams (20 grams x 2) of extra milk derived proteins daily compared with 7.5 grams (3.75 grams x 2) in the isocaloric placebo group. After each exercise session and ingestion of supplement the participants will record the level of exercise and amount of ingested supplement in a dairy. The compliance to the home-based exercises, nutritional supplements and vitamin D supplement is assessed also by interviewing the patients during each study visit. The intervention will last for 12 months. The nutritional contents of the protein supplement and isocaloric placebo are shown in Table 2. All supplements are manufactured and provided by Valio Ltd, Helsinki, Finland.

**Table 2:** Nutritional content of the supplements

	**Protein supplement 100 g**	**Isocaloric placebo 100 g**
Proteins (of which whey), g	8.3 (4.0)	1.0 (0.2)
Carbohydrates (of which lactose), g	7.0 (0.0)	14.0 (0.0)
Fat, %	1.0	1.0
Energy, kcal	70.0	70.0
Vitamin D, ug	1.0	0.2

All supplements are manufactured and provided by Valio Ltd, Helsinki, Finland.

### Outcome measurements

The examinations are performed at 0, 6 and 12 months. In addition to this, follow-up data are also collected from registries at 24 months.

Primary outcome measures are:

1. The 6- and 12-month changes in physical performance according to mean SPPB [21] and CSPPS [22,23] scores

2. Changes in muscle functions according to hand grip strength [17], gait speed [18], balance [19], chair stand test [24], two-minute step test [25]

Secondary outcome measures are:

1. Compliance to supplementation and home based exercises

2. Patient reported benefits and adverse effects

3. Changes in nutrition according to MNA [26], diet quality questionnaire [27], and dietary records [28]

4. Changes in body composition according to BMI and segmental BIS measurements [6,19]

5. Changes in cognition according to MMSE [20], verbal fluency [29,30] and clock drawing test [31], and CDR sum of boxes [35]

6. Changes in HrQOL measures according to SF-36 [32] and 15D [33]

7. Number of falls

8. Serum and plasma markers of inflammation, anabolism, glycemic control and renal function

9. Use of health care (number of hospitalization/follow-up time) and social services (number of institutionalization/follow-up time) during a 24 month follow-up

10. Mortality during a 24 month follow-up

### Statistical analyses

Required sample size calculation (PS: Power and Sample Size Calculations version 3.0.43, Nashville, TN, USA) is based upon the change in SPPB. A change of 0.5 in SPPB score has been defined as minimally important and change of 1.0 as substantially important. With a standard deviation (SD) of 2.0 for SPPB, a type I error rate of 5%, a power of 80%, and a drop-out rate of 25%, we will need approximately 80 patients in each group to detect a substantial change in SPPB, when the effects of protein supplementation are compared against no additional supplementation or isocaloric placebo with a Student’s t-test independently. Multiple comparison will be performed by Dunnett’s test [36]. The final sample size may differ slightly from this figure, because we will recruit participants from the entire 75 years and older population living in Porvoo, Finland, and the aim is to include as many as possible from those answering the postal questionnaire.

The statistical analyses will be performed by PASW statistics 18.0.3 (SPSS Inc. Chicago, IL, USA). In the baseline findings, for the continuous variables, descriptive values will be expressed by means with SD and medians with range. For the variables with a normal (Gaussian) distribution, statistical comparisons between the groups will be made by using an analysis of variance test. If the variables have a non-normal distribution or ordinal level, statistical comparison between groups will be performed with an independent samples Kruskal-Wallis test. Measures with a discrete distribution will be expressed as percentages (%) and analyzed by X^2^ or Fisher’s exact test when appropriate.

The results will be analyzed according to intention to treat. For the most important outcome parameters estimation of confidence interval (95%) will be used in addition to testing.

## Discussion

This rigorous randomized controlled trial will test whether and to what extent protein supplementation is able to maintain physical performance in community dwelling older people with sarcopenia. In addition to protein supplementation, the systematic use of simple and evidence-based methods to maintain muscle health in old age including home-based exercise, vitamin D supplementation and protein rich foods are promoted among all participants. The intervention is pragmatic in nature, and is performed in close collaboration with the municipal health care services. Thus, if the intervention proves to be effective, it can be implemented easily.

The strength of this study is its pragmatic nature [37]. Thus, the findings should be applicable in real life. The target population is frail and vulnerable and, according to our previous studies, is at risk of adverse outcomes such as physical disability and poor quality of life and death. Thus, ceiling effect is not easily reached in the primary endpoint. Furthermore, the limited exclusion criteria and portable nature of all study procedures allow the inclusion those disabled older people that are otherwise often not included in clinical trials.

However, there are also potential limitations in this study. First, the population is old and frail with many comorbidities, and, thus, vulnerable to competing causes of complications and deaths. This may decrease the power of the study. The second challenge relates to a sufficient difference to be attained between the groups with our intervention because nutritional supplementation may diminish the intake of regular food. Furthermore, the long-term adherence of patients to single taste supplements and unsupervised home-based exercises is also a challenge to this study.

To our knowledge, this is the first large scale randomized controlled trial among community dwelling older people with sarcopenia that focuses on the effects of protein supplementation on physical performance and several aspects of muscle health, nutritional status, QoL, cognition and hospitalizations.

## Trial status

Recruiting was underway at time of manuscript submission.

## Abbreviations

BIA: Bioimpedance analysis; BIS: Bioimpedance spectroscopy; BMI: Body mass index; CDR: Clinical dementia rating scale; DEXA: Dual x-ray absorptiometry; DSM-BIA: Direct segmental multifrequency measurements; HrQOL: Health-related quality-of-life; MMSE: Minimental state examination; MNA: Mini nutritional assessment; QOL: Quality of life; SD: Standard deviation; SMI: Skeletal muscle index; SPPB: Short physical performance battery.

## Competing interests

Dr Mikko Björkman reports professional cooperation including lecturing fees from Valio Ltd. Dr Pitkälä reports having professional cooperation including lecturing fees from pharmaceutical and other health care companies (including Janssen-Cilag, Lundbeck, MSD Finland, Orion, Pfizer, Novartis, Nestle), and having participated in clinical trials funded by pharmaceutical companies. Merja Suominen, Dr Harriet Finne-Soveri, and Dr Reijo Tilvis have no competing interests.

## Authors’ contributions

MPB, MHS, KHP, F-SUH, and RST participated in study conception and design; MPB, MHS, KHP, F-SUH, and RST participated in acquisition of data, or analysis and interpretation of data; and MPB, MHS, KHP, F-SUH, and RST participated in drafting or critically revising the manuscript for important intellectual content. MPB had full access to all of the data in the study and takes responsibility for the integrity of the data and the accuracy of the data analysis. MPB is the guarantor. Valio Ltd. and other funders of this study will have no author contributions. All authors read and approved the final manuscript.
